# The Relationship Between the Shape of the Spine and the Width of Linea Alba in Children Aged 6–9 Years. Case-Control Study

**DOI:** 10.3389/fped.2022.839171

**Published:** 2022-05-04

**Authors:** Anna Zmyślna, Arkadiusz Żurawski, Tomasz Rosiński, Justyna Pogorzelska, Zbigniew Śliwiński, Grzegorz Śliwiński, Wojciech Kiebzak

**Affiliations:** ^1^Collegium Medicum, Institute of Health Sciences, Jan Kochanowski University of Kielce, Kielce, Poland; ^2^Swietokrzyskie Paediatrics Centre, Regional Hospital Complex in Kielce, Kielce, Poland; ^3^Multispecialist Hospital-Independent Public Health Care Centre in Zgorzelec, In-patient Rehabilitation Centre, Nowa Sól, Poland; ^4^TU Dresden, Faculty of Electrical and Computer Engineering, Institute of Biomedical Engineering, Dresden, Germany

**Keywords:** postural defects, linea alba, Diers system, Physiotherapy, children

## Abstract

**Introduction:**

Civilization development coupled with contemporary lifestyle leads to a systematic increase in postural disorders. An analysis of factors that may provoke postural disorders indicates that such a stimulus may be the diastasis of the rectus abdominis muscles. Moreover, abnormal activity of the rectus abdominis muscles may affect balance disorders through reduced spinal stabilization and disturbed body statics. There is an increase in body posture abnormalities between the ages of 6 and 9 related to new school duties.

**Purpose of the Study:**

The purpose of the study was to evaluate the relationship between the shape of the spine and the width of the linea alba in children aged 6–9 years.

**Material and Method:**

The study was designed to evaluate parameters determining the shape of the spine, and the width of the linea alba in healthy children aged 6–9 years. The study participants were divided into two groups based on the width of the linea alba. The study group with the width of the linea alba >10 mm and the control group with the width of the linea alba ≤ 10 mm. The study group were included 37 children and the control group 24 children. The examination of the linea alba width was performed by a radiology specialist using a linear transducer and SAOTE- My Lab Classc-type ultrasound at rest and during contraction of the rectus abdominis muscles. Parameters describing the shape of the spine were measured using the Diers Formetric 4D system: trunk inclination, trunk imbalance, pelvis tilt, pelvic torsion, kyphotic apex, lordotic apex, kyphotic angle, lordotic angle, rotation angle, trunk torsion, lateral deviation. The obtained results were statistically analyzed using a Paired *t* test for comparison of differences between the results in the study and control groups and Pearson's test to assess the correlation between the width of the linea alba and parameters describing spinal alignment.

**Results:**

In both groups, the parameters describing the shape of the spine did not differ from the norms accepted as typical for the age norm. The only statistically significant difference between the study and control group concerned the trunk inclination, which was negative in the study group, signifying a shift of the entire spine axis backwards beyond the vertical.

**Conclusions:**

There is a correlation between the shape of spine and the width of the linea alba in terms of selected parameters determining the body posture in the sagittal plane, which concern: the position of the lumbar lordotic apex, trunk inclination and the depth of the lumbar lordotic angle. The width of the linea alba is not explicitly related to abnormalities of pelvic and spinal alignment in the frontal and transverse planes.

## Introduction

Civilization development coupled with contemporary lifestyle leads to a systematic increase in postural disorders (1). In addition, the abovementioned increase in disorders, and thus deterioration of health in children and adolescents, is influenced by a decrease in physical activity and by exceeding the organism's demand for energy (2). Our own observations, confirmed by the conclusions of other authors, indicate that postural defects are often a consequence of unhealthy daily habits. Thus, the habitual incorrect adoption of a specific body posture affects the formation and consolidation of a faulty body position (3–5). According to some authors, between the ages of 6 and 9 years, there are visible posture abnormalities related to new school duties. It is believed that prolonged sitting at the desk, asymmetrical carrying of backpacks and a small amount of activity oriented on maintaining good posture contribute to the development of musculoskeletal disorders. The abnormalities mainly concern the shape of the spine, the asymmetry of the torso and the work of the muscles (6, 7). Ignoring postural defects at an early stage of development may contribute negatively to the occurrence of health complications at a later stage.

In most children, postural dysfunction (8, 9), which often becomes apparent in the sagittal plane (2, 10–12), is considered to be the cause of these disorders.

An analysis of factors that may provoke postural disorders indicates that such a stimulus may be the diastasis of the rectus abdominis muscles (13–15). Moreover, abnormal activity of the rectus abdominis muscles (RA) may affect balance disorders through reduced spinal stabilization and disturbed body statics. In this perspective, it should be emphasized that correct tension of abdominal muscles enables to maintain the organs in the right place and to exert proper pressure on the lumbar region of the spine. This has a preventive role in deepening the lumbar lordosis and increasing the anterior tilt of the pelvis (13–15) and allows the body to maintain a correct posture. From a functional point of view, the linea alba plays an essential part in maintaining the stability of the body posture.

Its tension is regulated by the pyramidal muscles and the rectus abdominis muscles (16). The length of the linea alba (LA) in an adult is ~33 cm, while its width is at least 10 mm (14). The linea alba is running from the xiphoid process of the sternum to the superior pubic ligament. In the structure of linea alba, there are three different zones of fiber orientation: the lamina fibrae obliquae, the lamina fibrae transversae and the small lamina fibrae irregularium. The transverse fibers act as a counterpart to the intraabdominal pressure, whereas the oblique fiber are involved mainly in movements of the trunk (17).

Variability of posture, diversity of body types and variety of standards describing it make it difficult to distinguish between a normal and abnormal posture. Nowadays, a precise and non-invasive examination using computer diagnostic techniques is gaining popularity. Formetric measurement technology allows scanning the topography of the body surface without radiation or the use of markers. Anatomical landmarks, spinous process alignment and vertebral rotations are automatically detected (18–22).

## Purpose Of The Study

The purpose of the study was to evaluate the relationship between the shape of the spine and the width of the linea alba in children aged 6–9 years.

## Materials and Methods

### Study Design

The study was designed to evaluate parameters determining the shape of the spine, and the width of the linea alba in healthy children aged 6–9 years.

The study participants were divided into two groups based on the width of the linea alba.

- The study group with the width of the linea alba >10 mm
- The control group with the width of the linea alba ≤ 10 mm

The study group consisted of 37 patients of Physiotherapy of the Swietokrzyskie Pediatrics Center in Kielce. The study group included 6 children aged 6 years, 12 children aged 7 years, 9 children aged 8 years and 10 children aged 9 years.

The control group consisted of 24 patients of Physiotherapy of the Świȩtokrzyskie Pediatrics Center in Kielce. The study group included 4 children aged 6 years, 6 children aged 7 years, 6 children aged 8 years, and 8 children aged 9 years.

The sample size is not representative of the total population of children with diastasis linea alba.

The parameters were assessed once during the prophylactic examination. The parameters describing the shape of the spine were obtained using digital photogrammetry. The width of the linea alba in its particular sections was determined with the use of ultrasonography.

Consent of the Bioethics Committee of Jan Kochanowski University in Kielce No. 45/2028 was obtained for the study.

### Participants

A total of 61 children (31 boys and 29 girls) were included in the study. Their age ranged from 6 to 9 years.

The level of anthropometric indicators (height, weight, BMI) among the examined children was similar (Tables 1, 2).

**Table 1:** Anthropometric indicators among study group.

**Study group**
	**Average**	**Mediana**	**Minimum**	**Maximum**	**Variance**	**Standard deviation**
Age	7.62162	8	6	9	1.13063	1.063311
Weight [kg]	26.35135	25	18	40	30.28979	5.503616
Height [m]	1.23946	1.26	1.04	1.49	0.01153	0.107366
BMI	17.05081	16.64201	13.6048	22.27668	4.63867	2.153756

**Table 2:** Anthropometric indicators among control group.

**Control group**
	**Average**	**Mediana**	**Minimum**	**Maximum**	**Variance**	**Standard deviation**
Age	7.75	8	6	9	1.23913	1.113162
Weight [kg]	29.91667	28.5	22	44	32.16667	5.671567
Height [m]	1.30458	1,285	1,18	1.46	0.00376	0.061289
BMI	17.47931	16.92511	13.82128	22.27668	5.48774	2.342593

The history and medical examination did not reveal any coexisting conditions that could affect the analyzed parameters.

Inclusion criteria:

- Age 6–9 years;
- No comorbidities that could affect the quality of the results;
- Declared consent of the legal guardian to participate in the study;
- Defect of posture regarding the enhancement of the abdomen;
- Normal body weight;
- Good overall health.

Exclusion criteria:

- presence of comorbidities that may contribute to body axis abnormalities
- lack of consent from the legal guardian to participate in the study such as Scheuermann's disease, genetic conditions such as Beckwith-Wiedemann syndrome, or metabolic disease obese and significantly underweight children.

No data are available in the literature regarding norms of the linea alba size in children. The norm values of this parameter for adults are also a matter of dispute (14). Due to the lack of values defining norms of the linea alba width for children, the authors assumed the minimum width of the linea alba of 10 mm in adults to be the anatomical norm (1).

### Course of the Study

#### Ultrasound Examination

The examination of the linea alba width was performed by a radiology specialist using a linear transducer and SAOTE- My Lab Classc-type ultrasound at rest and during contraction of the rectus abdominis muscles.

Measurement of the width of the linea alba at rest:

A. 3 cm above the navel
B. At the level of the navel
C. 2 cm below the navel

Measurement of the width of the linea alba during contraction of the rectus abdominis muscles:

D. 3 cm above the navel
E. At the level of the navel

The width of the linea alba during contraction of the rectus abdominis muscles at a point 2 cm below the navel was not assessed in children due to technical difficulties in conducting this measurement.

#### Assessment of Shape of Spine

**Figure 1:**
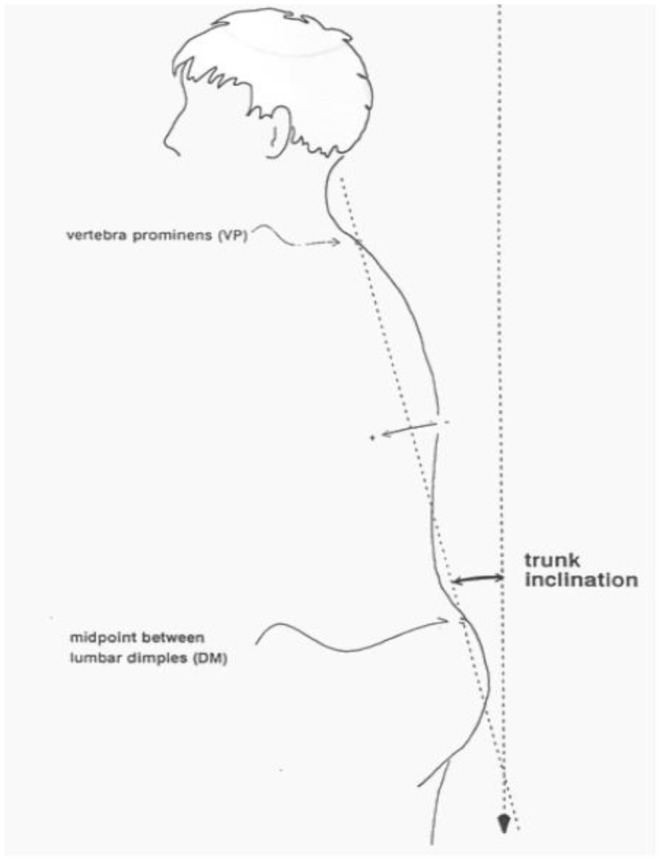
Trunk inclination.

**Figure 2:**
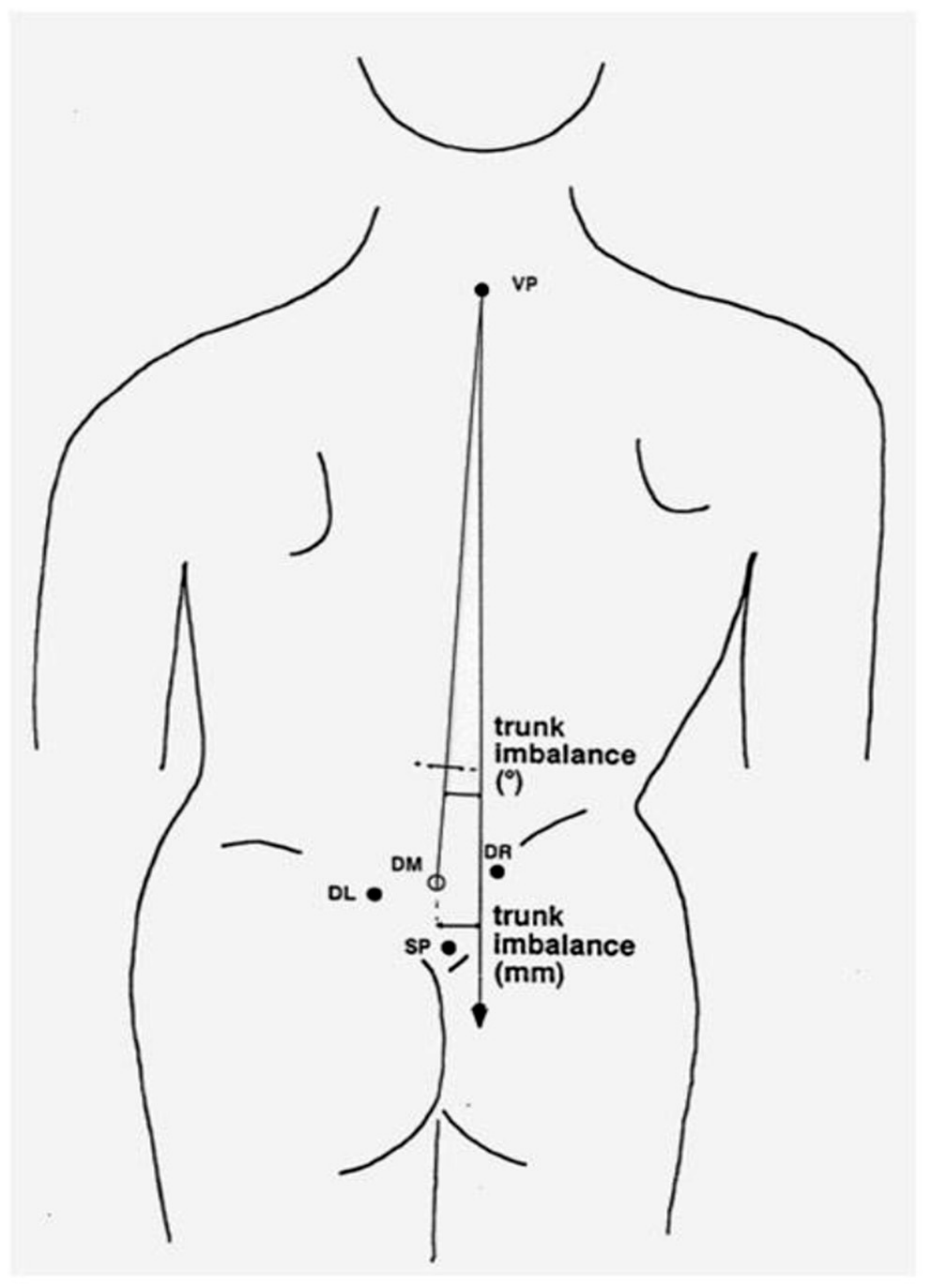
Trunk imbalance.

**Figure 3:**
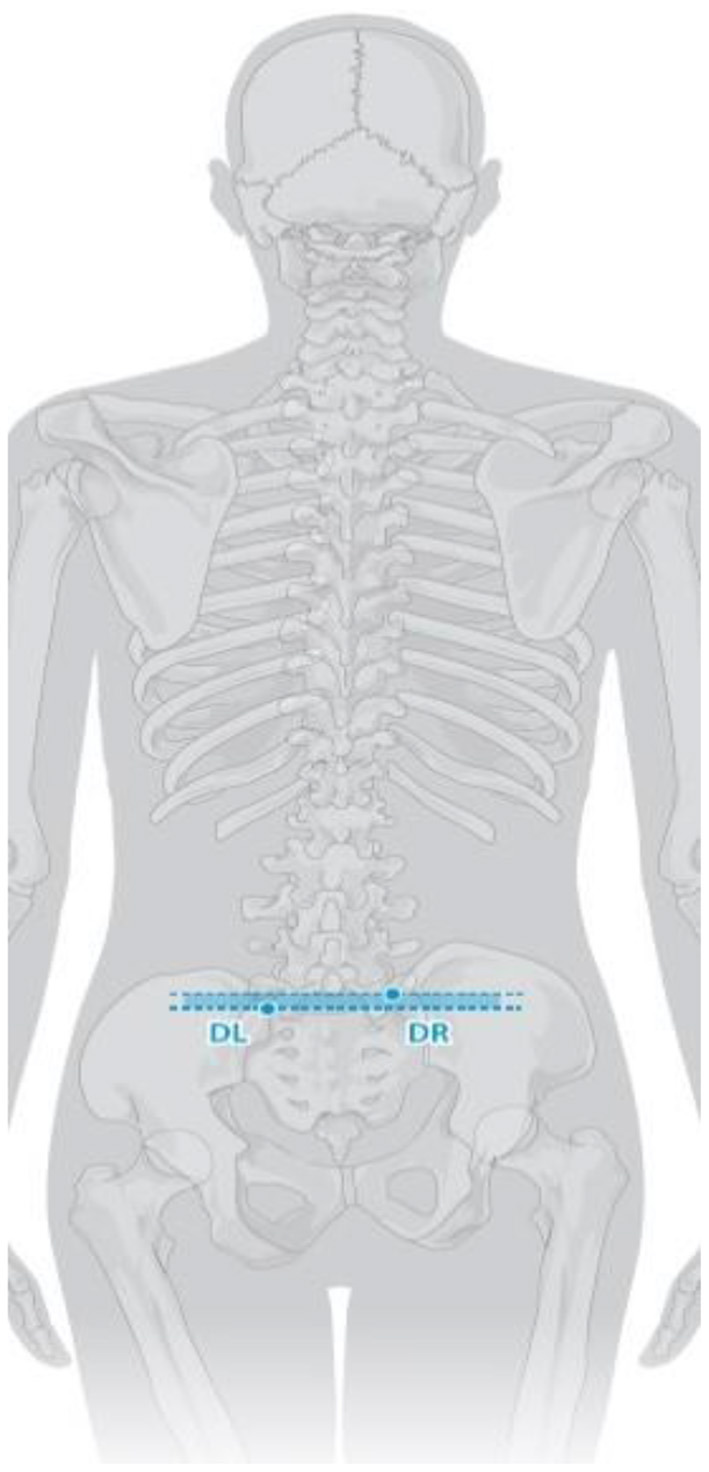
Pelvis tilt.

**Figure 4:**
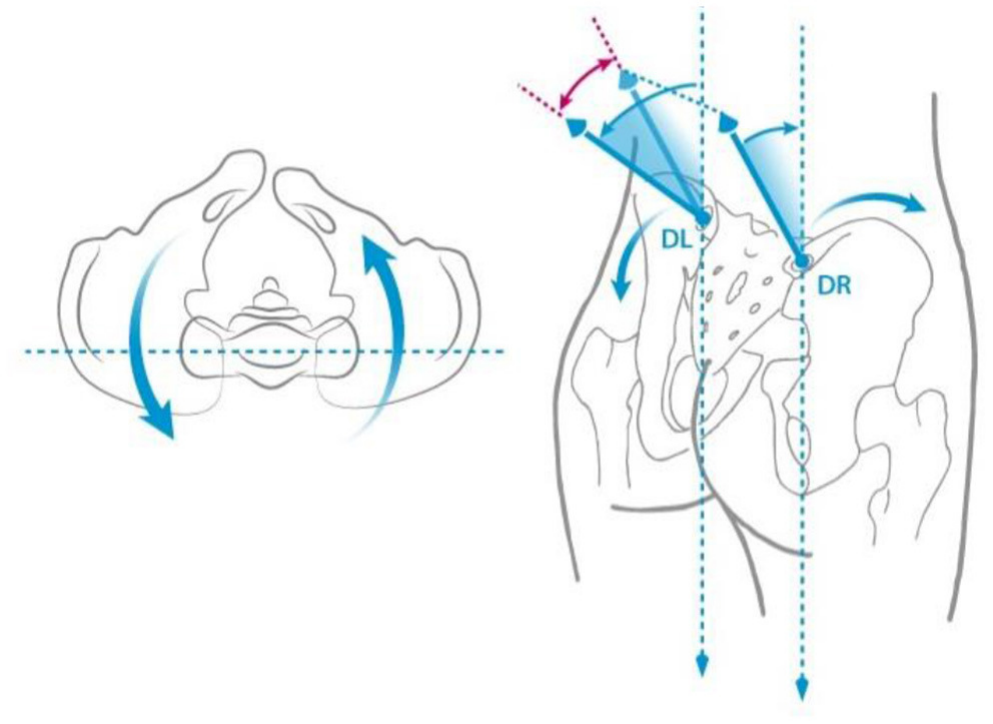
Pelvis torsion.

**Figure 5:**
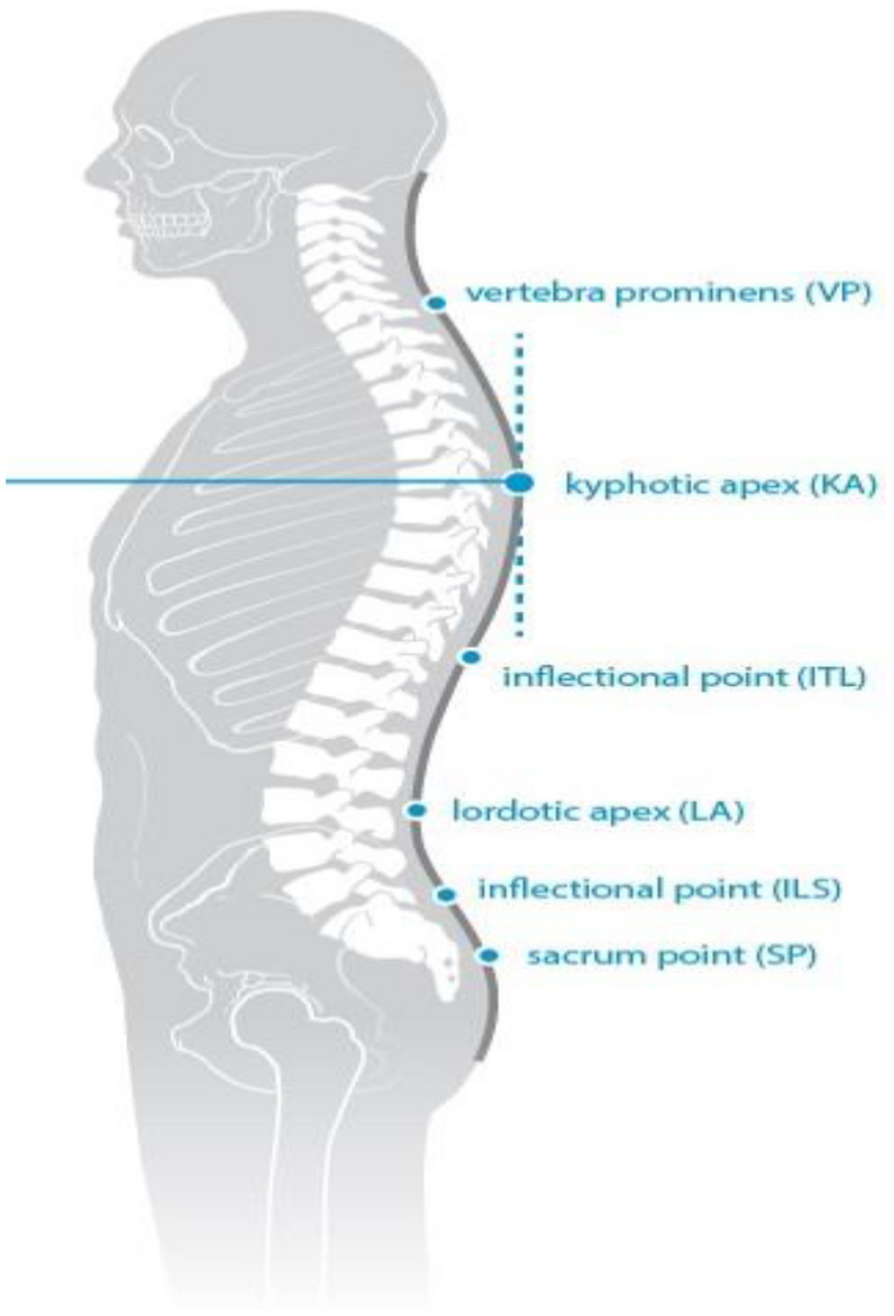
Kyphotic apex.

**Figure 6:**
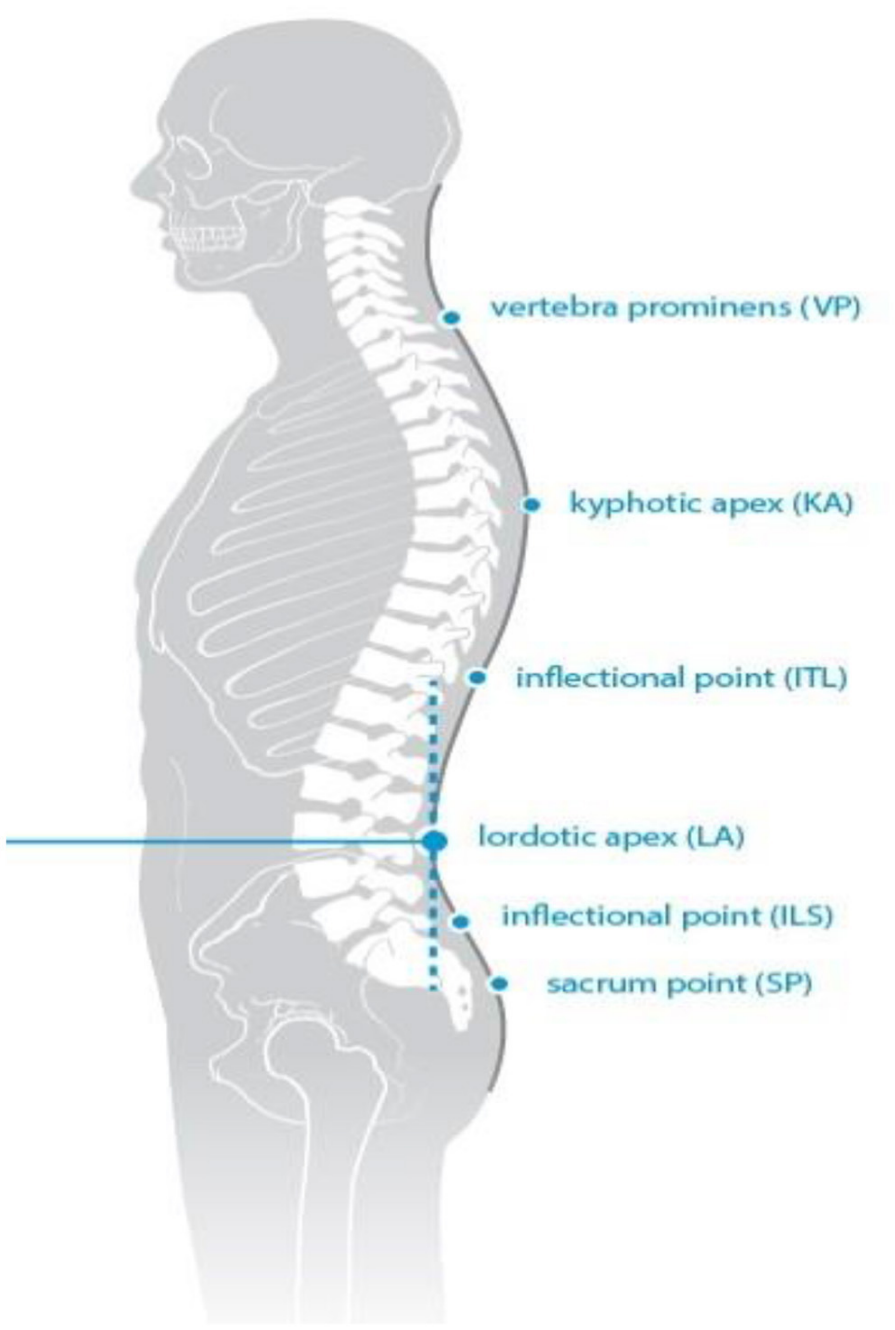
Lordotic apex.

**Figure 7:**
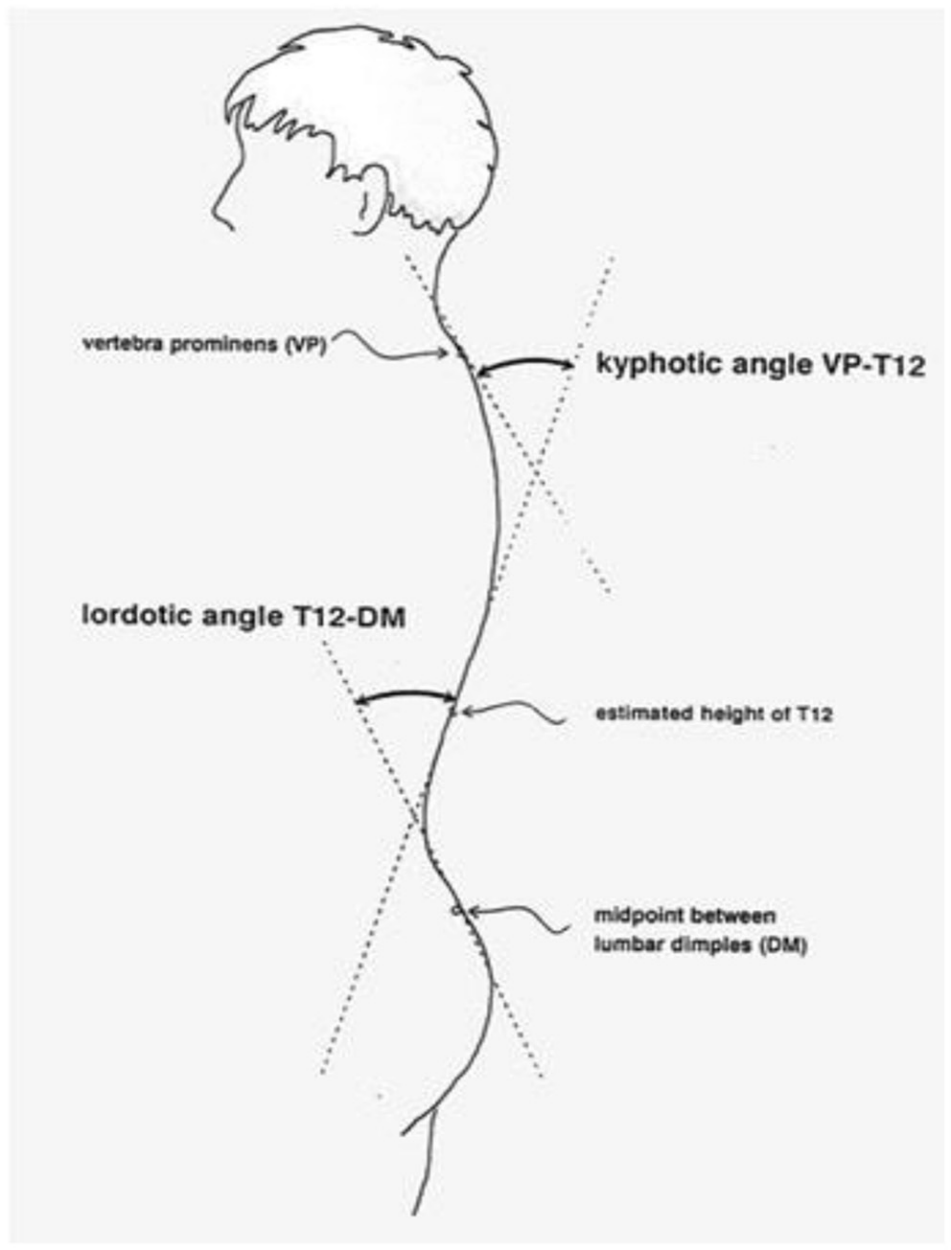
Kyphotic angle.

**Figure 8:**
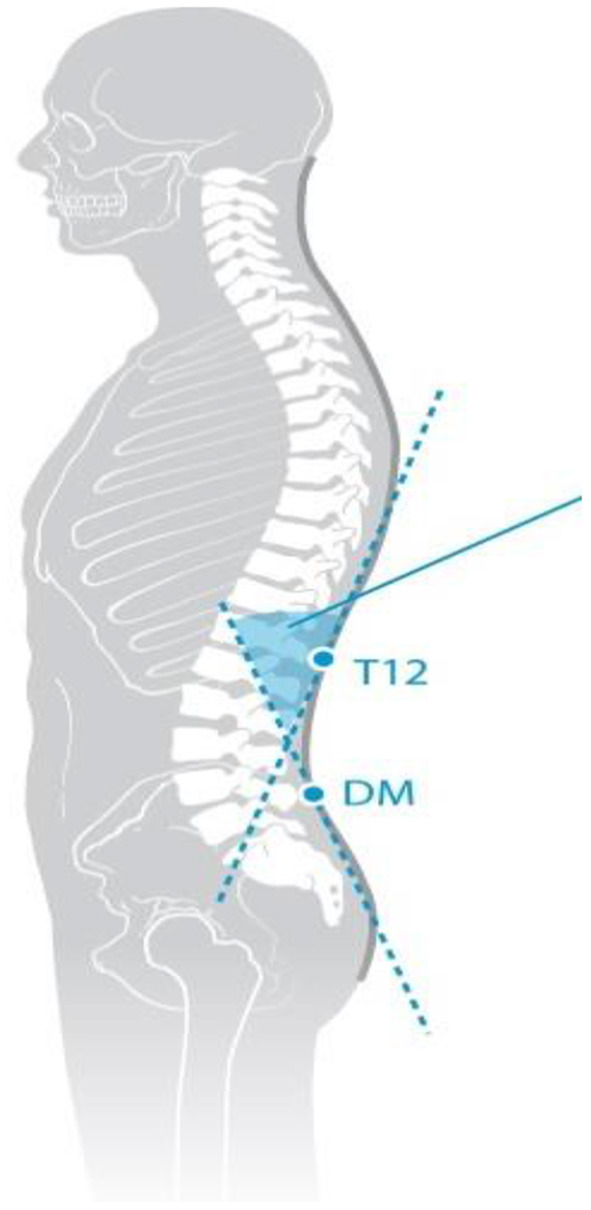
Lordotic angle.

**Figure 9:**
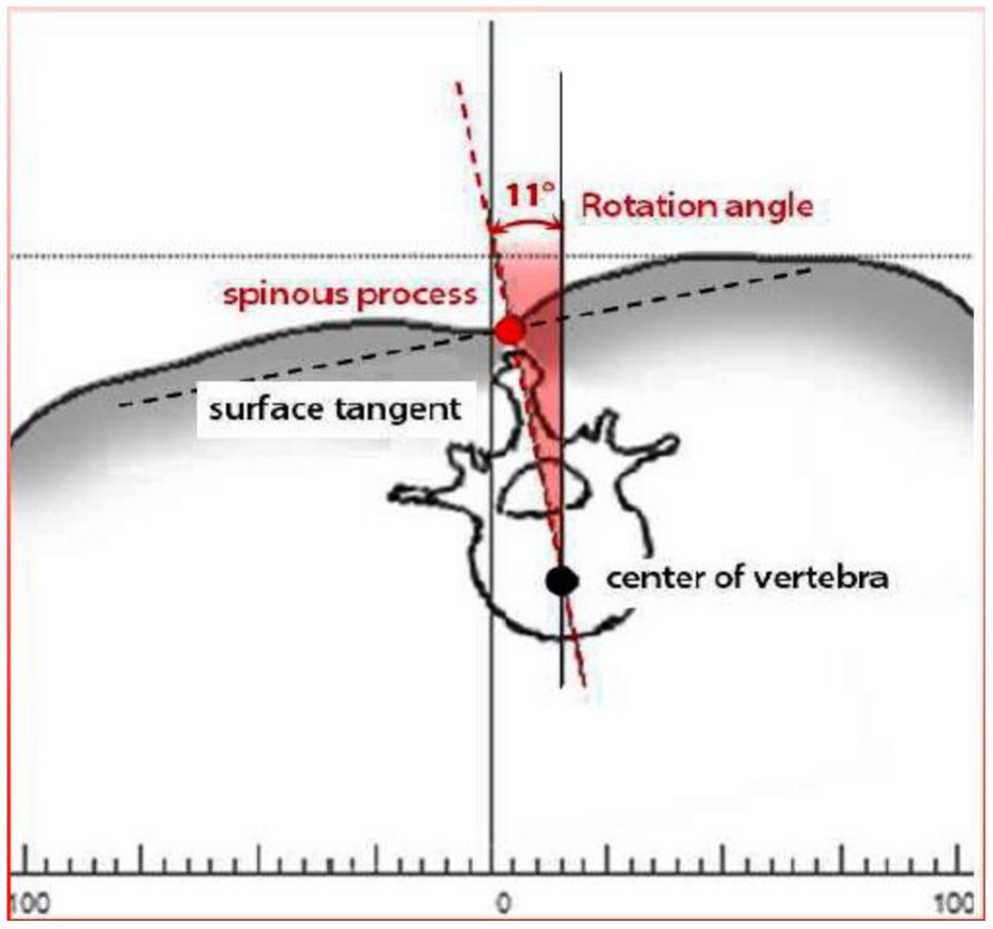
Rotation angle.

**Figure 10:**
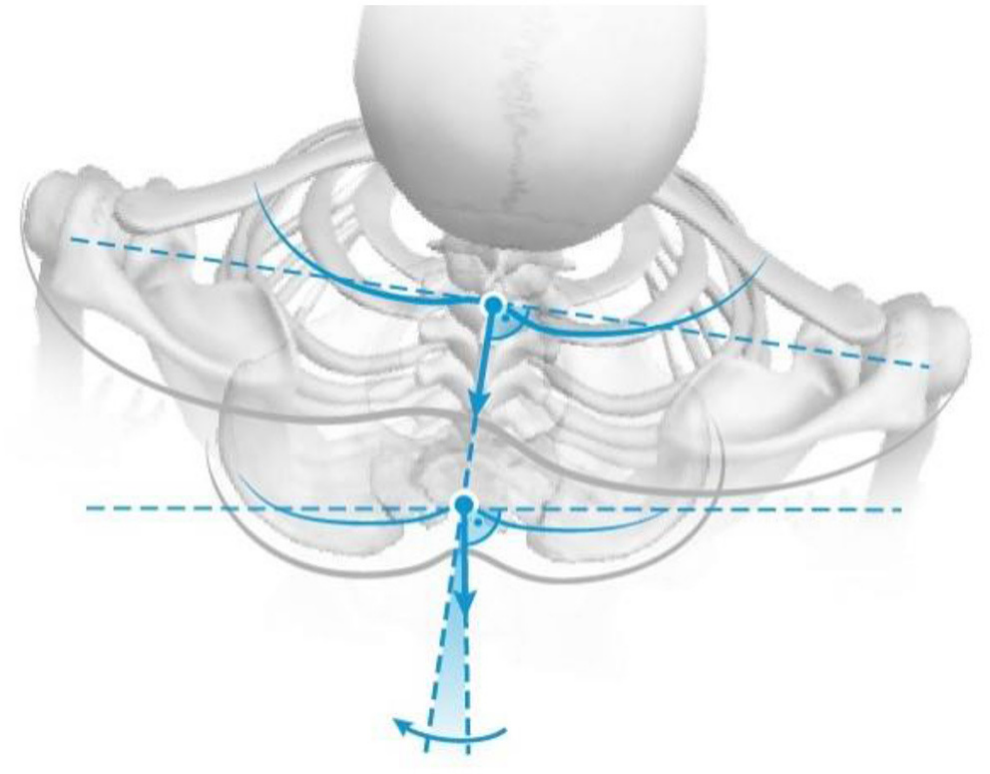
Trunk torsion.

**Figure 11:**
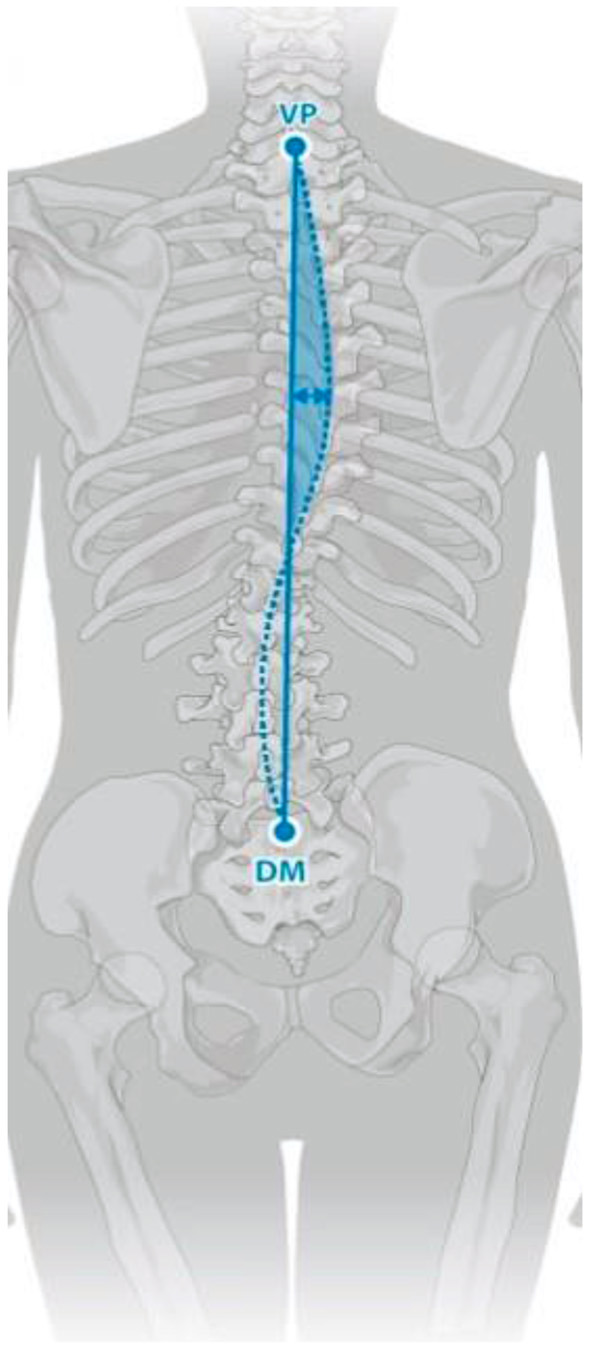
Lateral deviation.

Parameters describing the shape of the spine were measured using the Diers Formetric 4D system. The system acquires parameters to determine posture, back and spine surfaces. It allows to see diverse clinical parameters from an objective and quantitative static analysis of the body (18–23). The following parameters were assessed:

- Trunk inclination—the parameter is calculated in mm and represents the deviation of the line connecting VP (the 7th cervical vertebra) and DM (the center of the line joining the right and left anterior superior iliac spine) from the vertical in the sagittal plane (Figure 1).
- Trunk imbalance—the parameter is calculated in mm and represents the deviation of the line connecting VP and DM from the vertical in the frontal plane (Figure 2).
- Pelvis tilt—the parameter is measured in mm and refers to the difference in height between the lumbar dimples in relation to the horizontal plane (transversal section) (Figure 3).
- Pelvic torsion—the parameter is measured in [°] and is calculated from the reciprocal of the torsion of the normal planes at the lumbar dimples points (vertical component) (Figure 4).
- Kyphotic apex—posterior apex of the sagittal profile in the upper region, characterized by a vertical tangent (Figure 5).
- Lordotic apex—posterior apex of the sagittal profile in the lower region, characterized by a vertical tangent (Figure 6).
- Kyphotic angle—the parameter measured in °, this is the angle measured between VP and the estimated position of Th12 (Figure 7).
- Lordotic angle—the parameter measured in °, this is the angle measured between the estimated position of Th12 and DM (Figure 8).
- Rotation angle—the parameter measured in ° means the maximum rotation of the surface on the line of symmetry (Figure 9).
- Trunk torsion—the parameter is calculated from the reciprocal twist of the planes at DM and VP (Figure 10).
- Lateral deviation— the parameter is calculated in mm and represents the maximum deviation of the midline of the spine from the VP -DM line in the frontal plane (value at the top of curvature curve) (Figure 11).

### Statistical Analysis

The obtained results were statistically analyzed using a Paired *t* test for comparison of differences between the results in the study and control groups and Pearson's test to assess the correlation between the width of the linea alba and parameters describing spinal alignment. The correlation was considered statistically significant at *p* < 0.05.

## Results

In both groups, the parameters describing the shape of the spine did not differ from the norms accepted as typical for the age norm (23) (Tables 3, 4).

**Table 3:** Variables analyzed in the study group.

**Variable**		**Descriptive statistics**			
	***N***	**Average**	**Minimum**	**Maximum**	**Standard deviation**
A	37	14.027	4.000	28.000	4.65168
B	37	3.297	0.000	16.000	4.61799
C	37	12.351	5.000	28.000	4.25060
D	37	11.081	4.000	27.000	5.26719
E	37	9.838	3.000	23.000	4.20657
F	37	2.324	0.000	14.000	4.26259
Trunk inclination	37	−4.676	−36.300	23.350	16.64832
Trunk imbalance	37	−4.182	−25.500	19.500	9.12077
Pelvis tilt	37	−0.041	−9.000	12.000	4.49286
Pelvic torsion	37	0.570	−6.490	9.800	3.00721
Kyphotic apex	37	−101.727	−153.210	−12.190	32.38426
Lordotic apex	37	−275.296	−422.650	−176.270	43.64524
Kyphotic angle	37	35.201	15.430	55.900	10.02382
Lordotic angle	37	29.270	6.000	52.560	11.92092
Rotation angle	37	4.593	1.470	9.890	1.98316
Trunk torsion	37	−0.747	−21.830	11.130	6.55998
Lateral deviation	37	4.133	0.440	10.260	2.23784

**Table 4:** Variables analyzed in the control group.

**Variable**	**Descriptive statistics**				
	***N***	**Average**	**Minimum**	**Maximum**	**Standard deviation**
A	24	5.896	0.000	11.000	2.57909
B	24	7.750	3.000	11.000	2.15184
C	24	0.167	0.000	2.000	0.56466
D	24	4.625	0.000	10.000	2.64678
E	24	5.896	0.000	11.000	2.64567
F	24	0.000	0.000	0.000	0.00000
Trunk inclination	24	1.430	−25.960	42.260	15.83776
Trunk imbalance	24	−4.648	−16.500	4.500	5.55857
Pelvis tilt	24	−0.474	−9.000	6.000	3.48431
Pelvic torsion	24	0.190	−4.570	5.140	2.72091
Kyphotic apex	24	−105.624	−152.090	−45.920	28.23539
Lordotic apex	24	−271.833	−331.310	−211.110	34.03374
Kyphotic angle	24	35.460	21.000	53.670	9.06694
Lordotic angle	24	33.876	20.530	54.280	7.49276
Rotation angle	24	4.832	1.210	10.180	2.54246
Trunk torsion	24	−0.186	−16.500	7.720	5.63733

The only statistically significant difference between the study and control group concerned the trunk imbalance, which was negative in the study group, signifying a shift of the entire spine axis backwards beyond the vertical (Table 5).

**Table 5:** Comparison of the tested parameters in the study and control group.

**Study group vs. Control group**	**Paired** ***t*** **test**										
	**Average**	**Average**	**T**	**df**	***p***	***N***	***N***	**Standard deviation**	**Standard deviation**	**Quotient F**	***p***
Trunk inclination (study group) vs. trunk inclination (control group)	−4.67568	1.430417	−1.42603	59	0.159129	37	24	16.64832	15.83776	1.104978	0.815542
Trunk imbalance (study group) vs. trunk imbalance (control group)	−4.18243	−4.64833	0.224307	59	0.823294	37	24	9.120765	5.558573	2.692377	0.014669
Pelvis tilt (study group) vs. pelvis tilt (control group)	−0.040541	−0.47375	0.400298	59	0.690382	37	24	4.492862	3.484315	1.66269	0.201698
Pelvic torsion (study group) vs. pelvic torsion (control group)	0.57027	0.19	0.500485	59	0.618596	37	24	3.007208	2.720913	1.221512	0.621749
Kyphotic apex (study group) vs. kyphotic apex (control group)	−101.727	−105.624	0.48221	59	0.631441	37	24	32.38426	28.23539	1.315468	0.493961
Lordotic apex (study group) vs. lordotic apex (control group)	−275.296	−271.833	−0.32889	59	0.743405	37	24	43.64524	34.03374	1.644578	0.211527
Kyphotic angle (study group) vs. kyphotic angle (control group)	35.20081	35.46	−0.10235	59	0.918826	37	24	10.02382	9.066941	1.222208	0.620711
Lordotic angle (study group) vs. lordotic angle (control group)	29.27027	33.87625	−1.68639	59	0.097003	37	24	11.92092	7.492761	2.531256	0.021673
Rotation angle (study group) vs. rotation angle (control group)	4.592973	4.831667	−0.4106	59	0.682856	37	24	1.983164	2.542456	1.643576	0.176958
Trunk torsion (study group) vs. trunk torsion (control group)	−0.746757	−0.18625	−0.34401	59	0.732063	37	24	6.559982	5.637334	1.354122	0.448365
Lateral deviation (study group) vs. lateral deviation (control group)	4.132703	3.205833	1.737435	59	0.087528	37	24	2.237836	1.670011	1.795633	0.142173

Correlations between the width of the linea alba and parameters describing posture in the study group were analyzed (Table 6). Nine statistically significant correlations were obtained, but they were of weak strength. They concerned relationships of the linea alba width with trunk inclination, the position of the lumbar lordotic apex, the depth of the thoracic kyphotic angle and the depth of the lumbar lordotic angle.

**Table 6:** Correlations between the linea alba width and parameters describing body posture in the study group.

**Variable**	**Correlations** ***p*** **< 0.05000 *N>* = 37 (Missing data were removed on a case-by-case basis)**					
	**A**	**B**	**C**	**D**	**E**	**F**
Trunk inclination	−0.112676	0.130503	−0.370781	0.008384	0.093479	−0.353528
Trunk imbalance	−0.098193	−0.018807	0.020726	−0.123936	−0.055411	0.098805
Pelvis tilt	0.251258	0.170947	0.267691	0.172692	0.070191	0.203043
Pelvic torsion	−0.141187	−0.073611	−0.307622	−0.038162	−0.011635	−0.354746
Kyphotic apex	0.037788	−0.247535	0.223897	−0.214208	−0.266756	0.204840
Lordotic apex	−0.219095	−0.308676	0.171668	−0.432927	−0.340759	0.163954
Kyphotic angle	0.157471	0.361884	0.010262	0.284204	0.411823	−0.008113
Lordotic angle	0.254027	0.175422	0.241337	0.350601	0.328363	0.257998
Rotation angle	−0.181008	0.228399	0.150373	−0.184044	0.157057	0.164511
Trunk torsion	0.233771	0.267357	0.144211	0.117727	0.101356	0.143358
Lateral deviation	−0.188987	−0.012192	−0.224226	−0.076067	−0.054837	−0.178485

Correlations between the width of the linea alba and parameters describing posture in the control group were analyzed (Table 7). Two statistically significant correlations of moderate strength were obtained. They concerned the relationship between the depth of lumbar lordosis and the width of the linea alba at the level of the navel at rest and during abdominal muscle tension.

**Table 7:** Correlations between the linea alba width and parameters describing body posture in the control group.

**Variable**	**A**	**B**	**C**	**D**	**E**
Trunk inclination	0.045553	0.004864	−0.140123	0.052305	−0.010214
Trunk imbalance	−0.264158	−0.245250	0.216004	−0.212821	−0.229070
Pelvis tilt	−0.016036	0.227070	−0.090715	0.036473	0.230852
Pelvic torsion	0.049937	0.234806	−0.046410	0.002143	0.318781
Kyphotic apex	−0.147227	0.016963	0.371796	−0.210773	0.084516
Lordotic apex	−0.208355	0.119822	0.222427	−0.268501	0.232216
Kyphotic angle	−0.019085	−0.257986	−0.230141	0.025382	−0.346612
Lordotic angle	−0.163330	−0.654746	−0.292314	−0.063751	−0.642664
Rotation angle	−0.078246	0.272426	−0.326677	0.045163	0.209097
Trunk torsion	−0.195635	−0.048019	0.107699	−0.267503	−0.055244
Lateral deviation	−0.161214	−0.041801	−0.071158	0.028452	−0.083944

## Discussion

Currently, the increasingly recognized postural disorders among children are particularly severe in the sagittal plane and characterized by an abnormal shape of the spine (24). These abnormalities often contribute to the disruption of muscle tone throughout the body (25). According to the law of tensegrity, symptoms of abnormal tension can be seen even at distant locations from their source (25). Own research has not shown a strong correlation between the width of the linea alba and the resting position of the pelvis and spine in the frontal and transverse planes, but only with the depth of lumbar lordosis and, to a lesser extent, the depth of thoracic kyphosis.

In own observations, a relationship between abdominal muscle work and lumbar spine alignment was noted. These findings may be related to the posterior shift of the body axis demonstrated in children with diastasis musculorum rectorum. The abdominal muscles are important stabilizers of the spine, and their poor coordination, visible in diastasis musculorum rectorum, may contribute to chronic lower spine pain (26). In their original study, Doubkova et al. demonstrated a relationship between diastasis musculorum rectorum and chronic spinal pain (26).

The data obtained in the test group showed that the posterior shift of the body axis contributes to the shift of lumbar lordotic apex, whose position, as shown by own research, correlates statistically significantly with the width of the linea alba in children in the study group and may cause overloading of spinal joints in this segment. Our own study also showed a difference in body posture, which was the extent of trunk inclination. In the study group, this parameter was negative, meaning shifting of the whole trunk backwards. Shifting the whole trunk backwards in relation to the body axis requires constant tension of abdominal muscles, necessary to maintain the vertical position. Similar tension should accompany lateral flexion (27), however, our own study does not confirm a correlation of the width of the linea alba with the position of the spine in the frontal plane.

Studies have also failed to show strong correlations of spinal and pelvic alignment with diastasis musculorum rectorum in a static position. According to Benjamin et al., diastasis musculorum rectorum may be associated with lumbo-pelvic instability and pelvic floor weakness (28). Furthermore, when abdominal pressure is elevated for a prolonged period of time, the linea alba dilates (14).

In the conducted study, a statistically significant correlation was identified between the width of the linea alba at the level of the navel and 3 cm above during tension of the rectus abdominis muscle and the position of the lumbar lordotic apex in children from the study group. According to the literature, abdominal muscles such as rectus abdominis, external oblique and internal oblique muscles belong to global stabilizers, which are not the only stabilizing system in this region of the body. There is also a system of local stabilizers in the L-S segment (29). Some authors report that diastasis musculorum rectorum and weakening of the connections between the abdominal muscles can lead to a reduction in abdominal pressure and pressure on the vertebrae supplied by the thoracic fascia. Over time, the center of gravity shifts toward the lower part along the spine, increasing the pressure on the intervertebral discs and causing pain (30).

In the present study, a correlation between the width of the linea alba and the depth of lumbar lordosis was demonstrated in both the study and control groups. Similar results were described by Yalfani et al. investigating the correlation of lumbar lordosis size and the width of the linea alba (31). The correlations observed in own study were statistically significant, but characterized by moderate strength. The strength of the mentioned correlations indicates the existence of at least one more factor affecting the depth of lumbar lordosis and it may be the efficiency of local stabilizers. However, this thesis requires further research.

It should be emphasized that the correlation between the width of the linea alba and the depth of lumbar lordosis is greater in the group where the linea alba had a smaller width. This may indicate the presence of complex dysfunctions in the stabilization system in persons with diastasis musculorum rectorum.

In our own study, in the study group the width of linea alba correlated with both the kyphotic angle and the lordotic angle, while in the control group this correlation occurred only in relation to the lumbar lordosis. Temel et al. describes significant changes in the size of thoracic kyphosis and lumbar lordosis in patients undergoing surgical reduction of diastasis musculorum rectorum confirming the influence of this structure on the shape of the spine in the sagittal plane (32).

## Limitations Of The Studies

It is not possible to determine the chronology of events on the basis of the cited studies, whether the dysfunction of the linea alba affects the change in the depth of lumbar lordosis through a change in abdominal muscle tone, or whether abdominal muscle disorders cause the rectus abdominis muscle diastasis and changes in the shape of the lower spine. The authors see a need for further research in this area. Further studies should investigate the contractile activity of individual abdominal muscles and be conducted on a larger population divided into body weight centile ranges.

## Clinical Implications

As shown in the presented results, there is a significant correlation between the width of the linea alba and the depth of lumbar lordosis, this relationship can be used in building a treatment procedure for patients with linea alba diastesis.

## Conclusions

1. There is a correlation between the shape of spine and the width of the linea alba in terms of selected parameters determining the body posture in the sagittal plane, which concern: the position of the lumbar lordotic apex, trunk inclination and the depth of the lumbar lordotic angle.
2. The width of the linea alba is not explicitly related to abnormalities of pelvic and spinal alignment in the frontal and transverse planes.

## Data Availability Statement

The raw data supporting the conclusions of this article will be made available by the authors, without undue reservation.

## Ethics Statement

The studies involving human participants were reviewed and approved by Consent of the Bioethics Committee of Jan Kochanowski University in Kielce No. 45/2018 was obtained for the study. Written informed consent to participate in this study was provided by the participants' legal guardian/next of kin.

## Author Contributions

AZm, AŻu, WK, and ZŚ contributed to conception and design of the study. AZm, AŻu, TR, and JP organized the database. AŻu performed the statistical analysis. AZm and AŻu wrote the first draft of the manuscript. AZm, AŻu, ZŚ, GŚ, and ZŚ wrote sections of the manuscript. All authors contributed to manuscript revision, read, and approved the submitted version.

## Funding

This project was financed under the program of the Minister of Science and Higher Education called Regional Initiative of Excellence in the years, project no 024/RID/2018/19, amount of financing 11 999 000,00 PLN. The funders had no influence on the way the research was conducted, the content of the publication or the final conclusions.

## Conflict of Interest

The authors declare that the research was conducted in the absence of any commercial or financial relationships that could be construed as a potential conflict of interest.

## Publisher's Note

All claims expressed in this article are solely those of the authors and do not necessarily represent those of their affiliated organizations, or those of the publisher, the editors and the reviewers. Any product that may be evaluated in this article, or claim that may be made by its manufacturer, is not guaranteed or endorsed by the publisher.
